# Mechanical and geometrical determinants of wall stress in abdominal aortic aneurysms: A computational study

**DOI:** 10.1371/journal.pone.0192032

**Published:** 2018-02-05

**Authors:** Dara Azar, Donya Ohadi, Alexander Rachev, John F. Eberth, Mark J. Uline, Tarek Shazly

**Affiliations:** 1 Biomedical Engineering Program, College of Engineering and Computing, University of South Carolina, Columbia, South Carolina, United States of America; 2 Department of Chemical Engineering, College of Engineering and Computing, University of South Carolina, Columbia, South Carolina, United States of America; 3 Institute of Mechanics, Bulgarian Academy of Sciences, Sofia, Bulgaria; 4 Department of Cell Biology and Anatomy, School of Medicine, University of South Carolina, Columbia, South Carolina, United States of America; 5 Department of Mechanical Engineering, College of Engineering and Computing, University of South Carolina, Columbia, South Carolina, United States of America; Worcester Polytechnic Institute, UNITED STATES

## Abstract

An aortic aneurysm (AA) is a focal dilatation of the aortic wall. Occurrence of AA rupture is an all too common event that is associated with high levels of patient morbidity and mortality. The decision to surgically intervene prior to AA rupture is made with recognition of significant procedural risks, and is primarily based on the maximal diameter and/or growth rate of the AA. Despite established thresholds for intervention, rupture occurs in a notable subset of patients exhibiting sub-critical maximal diameters and/or growth rates. Therefore, a pressing need remains to identify better predictors of rupture risk and ultimately integrate their measurement into clinical decision making. In this study, we use a series of finite element-based computational models that represent a range of plausible AA scenarios, and evaluate the relative sensitivity of wall stress to geometrical and mechanical properties of the aneurysmal tissue. Taken together, our findings encourage an expansion of geometrical parameters considered for rupture risk assessment, and provide perspective on the degree to which tissue mechanical properties may modulate peak stress values within aneurysmal tissue.

## Introduction

A significant manifestation of cardiovascular disease involves a regional dilation of the aorta termed an aortic aneurysm (AA) [1–16]. Diagnosed using ultrasonography, computed tomography, or magnetic resonance imaging, a segment of the aorta that is found to be greater than 50% larger than that of a healthy individual of the same sex and age is considered aneurysmal [17]. AA can arise in either the thoracic or abdominal sections, with current estimates that over a quarter-million new cases of AA occur each year in the United States alone. Thus, several million patients carry the diagnosis of AA, and unfortunately a significant portion of these patients will either die from rupture or morbidity arising from complex surgical/endovascular repairs.

While stimuli for AA genesis and progression can be diverse, wall rupture is ultimately a mechanical failure that occurs when intramural stresses exceed wall strength [18,19]. Intramural stresses generally increase with aneurysm growth and inherently depend on the applied loads, the geometry and location of the AA, and the mechanical properties of the aortic wall. Despite the multiple determinants of wall stress, a set-point point of 5.0–5.5 cm in diameter is the typical threshold for surgical intervention [5,14]. Endovascular or surgical repair of AA is not without significant costs and complications [1,3,6,14–16]. Therefore, any advancement in terms of assessing the risk of AA rupture would be of high clinical significance.

Computational modeling utilizing finite element analysis (FEA) is a well-established approach to predict wall stresses in the context of AA. Previous studies have shown that, in addition to maximum AA diameter, centerline tortuosity is a deterministic parameter in AA rupture risk [20,21]. Other studies have also suggested that indices of AA surface curvature and wall thickness impact rupture risk [22], primarily via correlation of rupture location with geometrical features of the AA [23]. The results of any FEA-based study are highly dependent on the employed material models and distinct domains in which wall stress is computed. A previous study that assessed ruptured and non-ruptured AA via computed values of the peak wall stress (PWS) demonstrated that incorporation of various levels of geometric complexity derived from computed tomography data could significantly impact obtained results [24]. Thus, if the modeling objective is to generate a patient-specific prediction of wall mechanics in the context of AA, it is likely that high-fidelity geometries and accurate material models would be required.

As opposed to generating patient-specific predictions of AA wall mechanics, the purpose of this study is to compare the isolated and synergistic effects of general geometric and mechanical properties on AA rupture potential in idealized scenarios. Although both contribute significantly to the wall stresses experienced by the AA under physiological loading, only the former is regularly considered in risk analysis with most interventional criteria focused on aneurysmal sac diameter measurements alone. We systematically explore certain geometrical characteristics descriptive of AA (i.e., location within the parent vessel, axial/circumferential extent, thickness, and tortuosity) which may significantly increase mural stress levels and thereby warrant an elevated risk status for the patient. While noninvasive measurement of AA mechanical properties is admittedly limited, we utilize established constitutive models and prior studies to provide reasonable estimates of baseline mechanical properties. Thus we explore the potential for the use of representative mechanical properties, in combination with diverse but accessible measurements of AA geometric properties, as a basis for risk evaluation [25–27].

## Methods

### Overview

A series of finite-element based computational models of human abdominal aorta that are based on classic continuum mechanics were developed to quantify the wall stress field in the context of abdominal aortic aneurysm (AAA) under normotensive conditions. The premise of our study is that while the deformed outer diameter of AAA is the clinical standard for estimating rupture risk, other AAA characteristics may be useful in predicting local elevations in wall stress. Computational parametric studies were designed to isolate the dependence of wall stress on aneurysm geometry/location and tissue mechanical properties; the interactive effects of select characteristics on wall stress were also analyzed. Two response variables were extracted from each simulation, namely the average and peak Von Mises stress within the aneurysmal region.

### Referent normal aortic geometry

**Fig 1:**
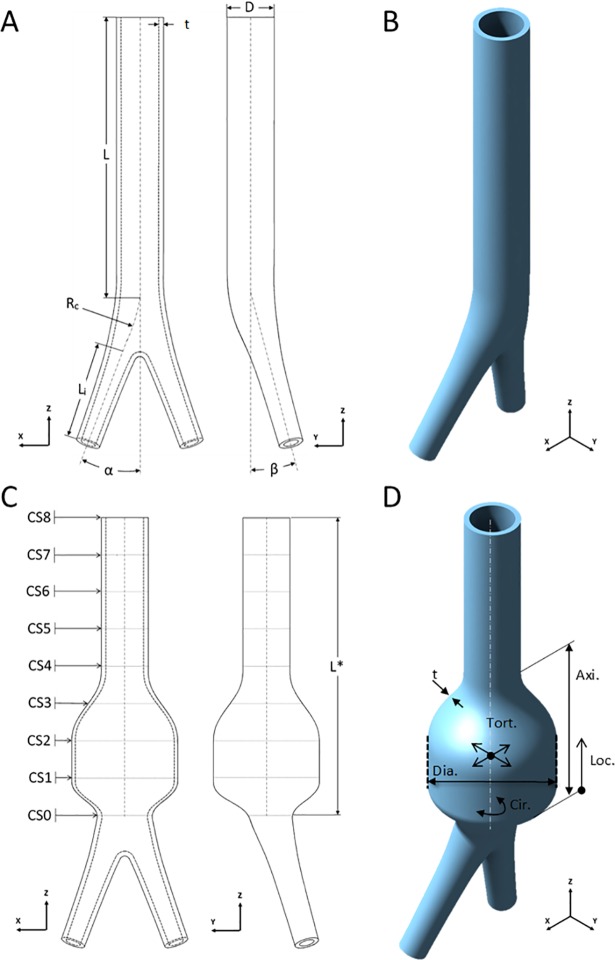
Referent normal aortic and baseline AAA geometries. (A) Referent normal aortic geometry in XZ- and YZ-planes; L is the length of abdominal aorta, D is the outer diameter, t is the thickness, R_c_ is the radius of curvature, L_i_ is the length of common iliac arteries, α and β are the take-off angle of the iliac arteries at the bifurcation and the angle between the abdominal aorta centerline and the plane formed by both iliac arteries, respectively. (B) An isometric view of the referent normal aortic geometry. (C) Baseline AAA geometry in XZ- and YZ-planes; L* is the length of geometrical variations field, CS0-8 are cross-sectional planes formed by dividing field of geometrical variations into eight sections longitudinally. (D) An isometric view of baseline AAA geometry and depiction of geometrical input variables; t is the thickness within the aneurysmal sac, Dia. is the maximum transverse outer diameter of the aneurysmal sac, Cir. depicts the circumferential extent of the aneurysmal sac, Tort. indicates the tortuosity in XZ and YZ-planes, Axi. and Loc. are the axial extent and the longitudinal location of aneurysmal sac, respectively.

The constructed geometry of the abdominal aorta and the aortic bifurcation were based on previous anatomical examinations [28]. Three-dimensional (3-D) geometrical models of the aorta (with and without AAA) were generated using CATIA V5R21 CAD software. All models entail a symmetric aorta-iliac bifurcation and common iliac arteries. The referent normal aortic geometry has dimensions: length (L) of 120 mm; outer diameter (D) of 20 mm; uniform wall thickness (t) of 2 mm (Fig 1A and 1B). The proximal diameter of the iliac arteries is 13 mm and gradually decreases to 10.3 mm over the considered length of these vessels (42 mm). The take-off angle of the iliac arteries at the bifurcation (α) and the angle between the aortic centerline and the plane formed by both iliac arteries (β), are 20° and 15°, respectively. The radii of curvature at the aorta-iliac junction is 50 mm on both sides.

### AAA geometric parameters

Geometric parameters used to characterize AAAs are defined with reference to cross-sectional planes (CS0 –CS8) spanning the longitudinal section of the aorta as follows: axial and circumferential extent of the AAA region, axial location of the AAA region, maximum transverse outer diameter of AAA region, local wall thickness and centerline tortuosity of the AAA region (Fig 1C and 1D). The baseline values and examined range for each geometric parameter (described below) were motivated by clinical observations of AAA (Table 1).

**Table 1:** Geometric parameter values for the baseline AAA (underlined) and associated parametric studies.

Axial Extent [mm]	Circumferential Extent [°]	Diameter^†^ [mm]	Location on Z-axis^1^ [mm]	Thickness [mm]	Tortuosity XZ-plane	Tortuosity YZ-plane
48	90	35	32	0.75	-1.175	-1.175
64^‡^	180	40	48	1.00	-1.05	-1.05
80	270	45	64	1.25	1.00	1.00
96	360	50		1.50	+1.05	+1.05
		55		1.75	+1.175	+1.175
		60		2.00		
		65				
		70				

† Maximum transverse diameter

1 Distance from the bottom-most cross-section (CS0) to the midway plane of aneurysmal sac in positive Z-direction

‡ Values underlined are geometrical input parameters defining the baseline AAA

### AAA geometry

The longitudinal length of the aorta considered for geometric variation (L*) is 128 mm and is defined by nine cross-sections (CS0 –CS8) that are parallel to XY-plane (Fig 1C). To impart geometric variations reflective of AAA and enable parametric computational studies, CS0 –CS8 were systematically manipulated and then closed splines connecting their perimeters were applied to define outer and inner surfaces. For the baseline AAA geometry, outer closed splines on CS1 and CS2 are circles (corresponding to a 360° circumferential extent) with a diameter of 45 mm; wall thickness smoothly decreases from its referent normal value of 2 mm to 1.50 mm on CS1 and CS2. All other CSs remained unchanged with respect to the previously generated referent normal aortic geometry, with exception of CS3, which was left free of constraints to produce a smooth evolution of surfaces. The midway plan of the baseline AAA sac is located 32 mm from CS0 (Fig 1C and 1D).

**Fig 2:**
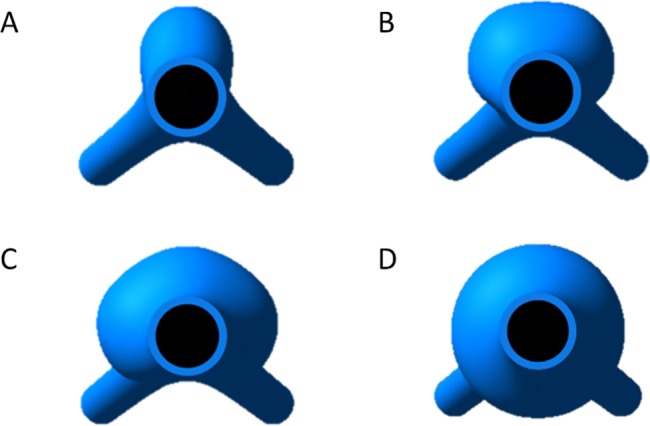
Circumferential (angular) extent of AAA bulging. Top views of a (A) 90° (B) 180°, (C) 270° sweep and (D) 360° sweep on anterior face of aneurysmal sac.

For sensitivity analyses, each geometrical parameter was varied in isolation over physiologically-relevant ranges as follows: AAA location via the center of the AAA sac along the Z-axis; wall thickness via the inner splines on CS1 and CS2; maximum AAA diameter via inner and outer splines on CS1 and CS2; tortuosity in XZ- and YZ- planes via the centers of inner and outer circles on CS1 and CS2; axial extent via the number of CS planes included within AAA sac; the circumferential extent of the AAA sac via progressive reduction of the anterior quarter of CS1 and CS2 by 90° (Fig 2).

### Mechanical properties of the referent normal aorta

The mechanical properties of aortic tissue were quantified using a previously developed structure-motivated constitutive model, where the strain energy function $\bar{\psi }$ is the sum of an isotropic term ${\bar{\psi }}_{iso}$ and anisotropic term ${\bar{\psi }}_{aniso}$,

$$\bar{\psi }={\bar{\psi }}_{iso}({\bar{I}}_{1})+{\bar{\psi }}_{aniso}({\bar{I}}_{4},{\bar{I}}_{6})$$ (1)

and ${\bar{I}}_{1}$, ${\bar{I}}_{4}$, and ${\bar{I}}_{6}$ are invariants of the right Cauchy-Green strain tensor [29,30]. A neo-Hookean material model is used for ${\bar{\psi }}_{iso}$ with the following analytical form,

$${\bar{\psi }}_{iso}({\bar{I}}_{1})=\frac{\mu }{2}({\bar{I}}_{1}-3)$$ (2)

where μ has the dimensions of stress and is the only material parameter [29]. ${\bar{\psi }}_{aniso}$ is the sum of two exponential functions that together describe the strain energy stored by two symmetrically-oriented families of collagen fibers, with fiber orientations defined by a helical angle of ±*φ* with respect to the longitudinal vessel axis. ${\bar{\psi }}_{aniso}$ has the following analytical form,

$${\bar{\psi }}_{aniso}({\bar{I}}_{4},{\bar{I}}_{6})=\frac{k_{1}}{2k_{2}}\sum_{i=4,6}\left\{{\exp[k_{2}({\bar{I}}_{i}-1)}^{2}]-1\right\}$$ (3)

where the invariants ${\bar{I}}_{4}$ and ${\bar{I}}_{6}$ each relate to one of the two fiber families. Moreover, *k*_*1*_ > 0 is a material parameter with units of stress and *k*_*2*_ > 0 is dimensionless [29]. The referent normal values for all material parameters (Table 2) are based on previous ex-vivo mechanical studies of aortic tissue [31].

**Table 2:** Mechanical properties for referent normal aortic tissue.

μ [MPa]	k_1_ [MPa]	k_2_	φ [°]
0.007	2.87	17.26	36.0

### Mechanical properties of aneurysmal tissue

Within the constitutive framework provided by (Eqs 1–3), Pierce et al. estimated material parameters for eight human AAA samples [32]. The fitted values for all material parameters informed our specification of baseline AAA as well as the examined ranges in parametric studies (Table 3). In all AAA simulations, collagen fiber angle *φ* was fixed at 45°and no fiber dispersion factor was applied.

**Table 3:** Mechanical property values for the baseline AAA (underlined) and associated parametric studies.

μ [MPa]	k_1_ [MPa]	k_2_	φ [°]
0.001	1.00	0.001	45.0 *
0.004	1.50	1	
0.009	2.00	30	
0.013^†^	2.74	70	
0.017	6.00	119.6	
0.040	15.00	350	
0.085	50.00	500	
0.100			

* Fiber angle of 45.0° was applied to all aneurysmal models

† Values underlined are material input parameters defining the baseline AAA case

### Meshing, boundary conditions and solving

Generated 3-D geometries were meshed using Altair HyperMesh v12.0 software package via first order hybrid tetrahedral elements. Tetrahedral elements were selected for meshing to avoid elemental collapse in model geometries with high curvature regions [33,34]. Mesh independency studies were performed on both the referent normal and baseline AAA geometries, wherein simulation results were deemed mesh-independent if additional mesh refinement led to a less than 4% change in both the peak and average von Mises stress. A total of 289,762 tetrahedral elements were required for mesh-independency, which set the minimum meshing threshold for all parametric studies. Boundary conditions were applied to the meshed geometries using a FEBio Software package pre-processor, PreView. A uniform pressure of 120 mmHg (0.016 MPa) was applied to the vessel inner surface via a gradual ramping up from zero pressure. The presented results thus refer to a stationary solution. A complete motion constraint (displacement and rotation) at all vessel initiation/termination surfaces were applied to facilitate solution convergence. All studies were performed with the full Newtonian solver settings in FEBio FEA open-source software [35].

### Post-processing of computational data

The direct output of all simulations (displacement vector field) was transformed using FEBio post-processor, PostView, with computed response variables including peak von Mises wall stress (PWS) and average von Mises wall stress (AWS) within the aneurysmal sac (or analogous location for the referent normal aorta). For calculation of AWS, obtained values were weighted by element size (area-weighted average stress).

## Results

### Wall stress in the referent normal aorta

**Fig 3:**
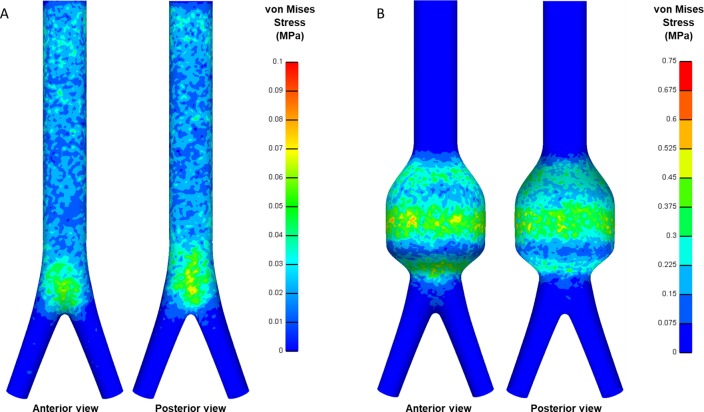
Predicted von Mises wall stress. Computed von Mises wall stress distributions for (A) referent normal aorta and (B) baseline AAA simulations.

Colorimetric surface plots (without gradient smoothing, anterior and posterior views) of the von Mises stress in the referent normal aorta reveal a relatively uniform wall stress distribution until the aortic bifurcation, with AWS of 0.013 MPa. A notable elevation in wall stress occurs at the aortic bifurcation, with a PWS of 0.091 MPa. Continuity of the von Mises stress field extending from the model boundaries qualitatively suggests that edge effects are minimized in the referent normal simulation (Fig 3A).

### Wall stress in AAA

Analogous surface plots of the von Mises stress field in the baseline AAA show a nonuniform wall stress distribution in comparison to the referent normal aorta. (Fig 3B). The AWS within the aneurysmal sac is 0.11 MPa, while a PWS of 0.76 MPa occurs near the sac center point. While stress values generally exhibit an order-of-magnitude increase in the baseline AAA as compared to the referent normal aorta, the PWS remains below the assumed ultimate strength (1 MPa) of aortic tissue [36] (discussed below).

#### Effects of AAA geometry

**Fig 4:**
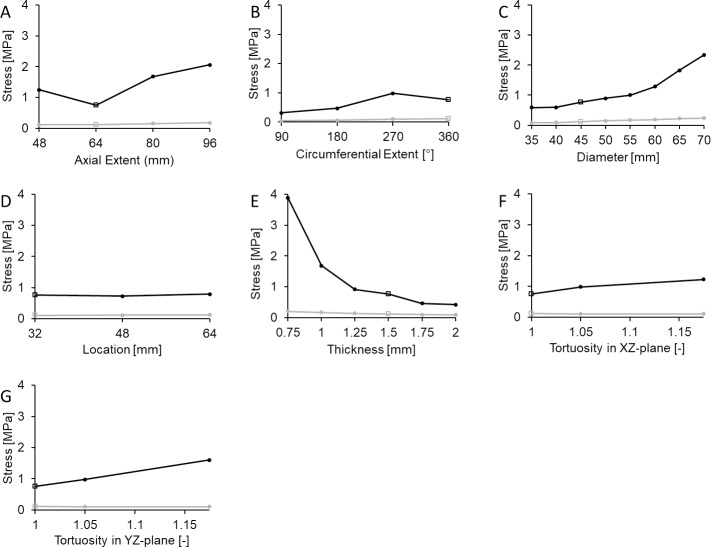
Effects of AAA geometrical properties. PWS (black) and AWS (grey) are computed over isolated variations of defined AAA geometrical properties, including (A) axial extent, (B) circumferential extent, (C) maximum sac diameter, (D) sac location, (E) wall thickness, (F) tortuosity in XZ plane, and (G) tortuosity in YZ plane. In all cases, hollow square markers indicate the baseline AAA values.

A series of simulations were performed under isolated variation of geometric parameters with respect to the baseline AAA model. AWS and PWS computed throughout these parametric studies reveal the relative sensitivity of wall mechanics to distinct morphological features (Fig 4A–4G). In all parametric sweeps, the percent variability in AWS over the examined range is reduced in comparison to that of PWS.

In terms of AAA axial extent, the largest value (96 mm) induces the greatest stress levels (Fig 4A). Interestingly, decreasing the axial extent with respect to the baseline AAA value (64 mm → 48 mm) also results in an increase in PWS; this is understood as a local consequence of increased sac curvature in proximity to the aortic bifurcation. Variation in AAA circumferential extent, as defined in this study, also has a non-monotonic effect on PWS (Fig 4B). In this case, a 270° circumferential extent of the sac causes an approximately 30% increase in PWS as compared to the baseline AAA case (circumferential extent of 360°); this is a consequence of sac asymmetry and the resultant increase in local curvature on the posterior sac surface. For all other examined scenarios, both PWS and AWS exhibit a monotonic response to isolated manipulation of the defined geometric parameters (Fig 4C–4G). Among these, the most deterministic parameters are clearly sac diameter (Fig 4C) and wall thickness (Fig 4E), for which maximal percent changes in PWS with respect to baseline AAA are 231% and 457%, respectively (Table 4).

**Table 4:** Sensitivity of PWS to geometrical and mechanical parameters characterizing AAA.

Parameter	Referent Normal	Baseline AAA	Range of Input Parameter	Percentage Change in PWS^2^
μ [MPa]	0.007	0.013	[0.001,0.1]	22.0%
k_1_ [MPa]	2.87	2.74	[1,50]	314%
k_2_	17.26	119	[0.001,500]	156%
Axial Extent [mm]	N/A	64	[48,96]	172%
Circumferential Extent [°]	N/A	360	[90,360]	88.1%
Diameter^†^ [mm]	20	45	[35,70]	231%
Location on Z-axis^1^ [mm]	N/A	32	[32,64]	9.06%
Thickness [mm]	2.0	1.50	[0.75,2.00]	457%
Tortuosity XZ-plane	1	1	[-1.175,1.175]	62.1%
Tortuosity YZ-plane	1	1	[-1.175,1.175]	112%

† Maximum transverse diameter

^1^ Distance from the bottom-most cross-section (CS0) to the midway plane of aneurysmal sac in positive Z-direction

^2^ Maximum percentage change over examined range with respect to baseline AAA value

#### Effects of AAA mechanical properties

**Fig 5:**
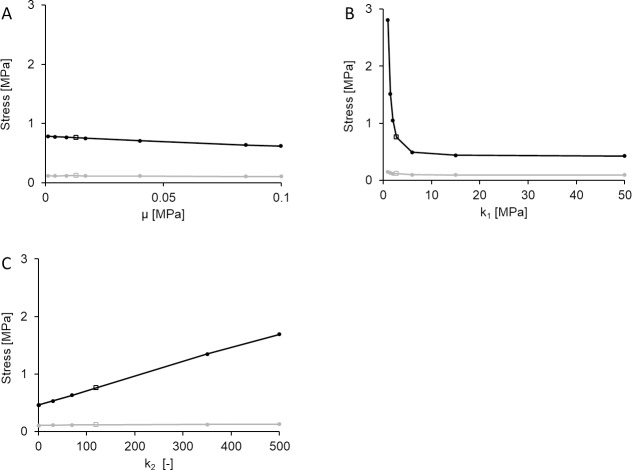
Effects of AAA mechanical properties. PWS (black) and AWS (grey) are computed over isolated variations of defined AAA mechanical properties, including (A) μ, (B) k_1_, and (C) k_2_. In all cases, hollow square markers indicate the baseline AAA values.

A second series of parametric studies entails isolated manipulations of the AAA mechanical properties with respect to the baseline AAA model. (Fig 5A–5C). As with geometrically-focused parametric sweeps, the percent variability in AWS over the examined range is reduced in comparison to that of PWS. PWS was notably more sensitive to mechanical properties associated with the anisotropic (as opposed to isotropic) component of the strain energy function (${\bar{\psi }}_{aniso}$), with *k*_*1*_ and *k*_*2*_ inducing a maximal percent changes in PWS with respect to baseline AAA of 314% and 156%, respectively (Table 4).

#### Interactive effects of AAA properties

**Fig 6:**
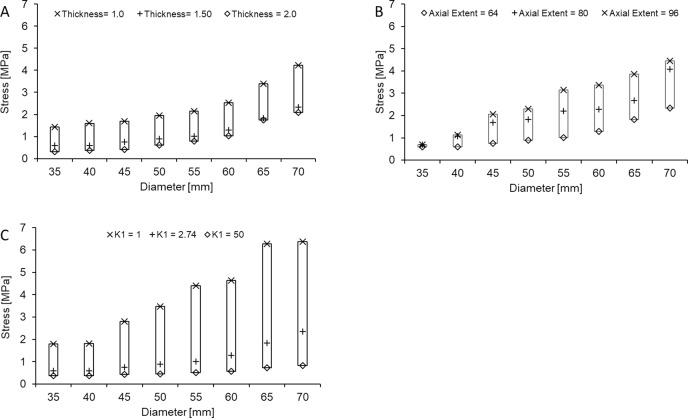
Interactive effects. PWS as a function of sac diameter and prescribed levels of (A) wall thickness, (B) axial extent, and (C) K_1_. The size of bars containing data points at a given diameter reflect the sensitivity of PWS to each parameter.

A third series of parametric studies examines the relative change in sensitivity of PWS to select AAA geometrical and mechanical properties with progressively increasing sac diameter (Fig 6). In the case of wall thickness, a 50% reduction (2 mm → 1 mm) leads to an increase in PWS that is approximately 1 MPa when the sac diameter is 35 mm, and 2 MPa when the sac diameter is 70 mm (Fig 6A). Comparatively, a 50% increase in AAA axial extent (64 mm → 96 mm) leads to an increase in PWS that is less than 0.02 MPa when the sac diameter is 35 mm, but over 2 MPa when the sac diameter is 70 mm (Fig 6B). An even more significant interactive effect occurs when diameter changes in tandem with AAA mechanical properties. When computed over the examined range of material parameter *K*_*1*_, the change in PWS is approximately 1.5 MPa at a sac diameter of 35 mm but increased to nearly 6 MPa at a sac diameter of 70 mm (Fig 6C).

## Discussion

The aim of this study is to apply finite element-based computational modeling to gain insight on the relative degree to which geometrical and mechanical properties of AAA determine the AWS and PWS within the aneurysmal sac. Potential interactive effects between select properties are also analyzed. Due to the inherent geometrical complexity, computational results pertaining to AAA wall mechanics are often transformed into scalar fields of von Mises stress and/or maximum principal stress to facilitate evaluation of the stress state [37]. In a similar computational model by Raghavan et al., the PWS computed for a referent normal aorta (diameter = 20 mm) was 0.12 MPa, which is close to our obtained value (0.091 MPa) [25]. As a benchmark for our baseline AAA model, we compared our results to findings by Roy et al. in which a model with similar geometry/properties exhibited a PWS of 0.76 MPa [38]. In the same study, they also considered patient-specific geometries and found a maximum principal stress of 0.79 MPa in a mid-sized AAA [38]. The reported values for PWS in these representative AAA scenarios are close to our findings in the baseline AAA model (PWS of 0.76 MPa). Finally, Rodriguez et al. developed a representative AAA model and computed a maximum principal wall stress of 0.79 MPa, which is close in value to the PWS obtain by us and others.

Aneurysmal sac rupture theoretically occurs when peak wall stress exceeds the ultimate strength of the vascular tissue. In line with this notion, it is not surprising that wall stress can predict sac rupture better maximum diameter [39,40]. To facilitate the quantification of rupture risk, numerous studies have sought to identify the ultimate tissue strength in AAA, wherein Giannoglu (2006) reported a median value of 1.23 MPa and Vande Geest et al. predicted values ranging from 0.54 MPa to 1.43 MPa [21,41]. Similarly, others suggest failure stresses on the order of 1MPa [36,42]. For the purposes of subsequent discussion, we consider the ultimate strength of the AAA wall to be 1 MPa.

Obtained results confirm that wall stress is highly sensitive to sac diameter (Fig 3C, Table 4), thereby supporting the established clinical approach of monitoring maximal sac diameter and its expansion rate for estimation of rupture risk. Our model predicts that as sac diameter reaches the standard clinical threshold for surgical intervention (5.0–5.5 cm), PWS is near/above the assumed ultimate strength of aortic tissue (1MPa). Moreover, increasing diameter above this threshold leads to a dramatic increase in PWS, supporting the need for immediate surgical intervention to prevent rupture.

Although many previous computational studies use uniform wall thickness to predict AAA mechanics [20,37,39,43–46], it is intuitively obvious that an isolated reduction in wall thickness will increase PWS within the aneurysmal sac. The choice to maintain a uniform wall thickness close to the baseline value is supported by some previous studies, in which AAA wall thickness was measured to be near its referent normal value [23,47]. Therefore, our predicted PWS response to varying wall thickness (Fig 4E) may not be applicable to all instances of AAA; rather, our results provide clinical motivation to monitor wall thickness as a precautionary measure. Indeed, a significant fraction (13%) of AAAs under 5 cm in diameter rupture [48], underscoring the need to identify additional risk factors beyond maximal sac diameter/expansion rate. Of the previous studies that did examine AAA wall thickness, Venkatasubramaniam et al. predicted that increasing/decreasing wall thickness by 25% leads to nearly a 20% decrease/increase in PWS, respectively [37]. In a multivariate study, Celi et al. (2010) showed that an AAA characterized by a relatively larger diameter and uniform thickness would exhibit reduced PWS compared to an AAA with a smaller diameter and reduced wall thickness [49]. In our study, PWS was the most sensitive to wall thickness within the explored parameter space, supporting its consideration as a key factor in clinical risk assessment.

Previous studies have demonstrated that as the axial extent of the AAA increases, PWS also increases [22,43]. While our results are in line with this expectation for axial extents exceeding the baseline AAA value (above 64mm), we also predict an increase in PWS at exceedingly low levels (48 mm). We speculate that this latter effect is a consequence on enhanced surface curvature in the distal portion of the AAA combined with the proximity to the aortic bifurcation. Indeed, Venkatasubramaniam et al. concluded that overall shape and sac asymmetry are significant determinants of AAA wall stress [37]. Other studies have developed various methods to interrelate sac asymmetry to wall stress levels, and in all cases concluded a positive correlation between the two [43,50–52].

AAA asymmetry can be magnified by increased tortuosity, which is a recognized geometric determinant of not only wall stress but also intra-aneurysmal flow patterns and resultant flow-induced shear stresses [20,21,43,53–58]. In our parametric sweeps, the applied tortuosity variation in either the XZ or YZ planes was modest in comparison to these and other previous studies due to the increased complexity of surface assignment. Nevertheless, PWS exceeding the tissue ultimate strength was observed within our examined range (Fig 4F and 4G). Although not realized in this study, we expect that additional increase of AAA tortuosity would lead to even greater PWS values.

Despite containing parametric sweeps focused on AAA mechanical properties within reported ranges for AAA tissue, it is important to note that isolated property manipulation in this sense may create a scenario (set of mechanical properties) that is not reflective of aneurysmal (or referent normal) tissue. Nevertheless, computed changes in AWS and PWS compared to those observed with geometric sweeps enable qualitative speculation on the relative impact of geometrical versus mechanical properties on AAA mechanics. Based on the notable percent change in PWS (314%) predicted for variation of *k*_*1*_, we conclude that material and geometrical properties are potentially equally deterministic of PWS.

### Study limitations

The conclusions drawn in this study should be considered along with some study limitations. Firstly, all simulations were performed under normotensive conditions, although some AAA patients often also present hypertension. Clearly the application of higher values of pressure in our model (to simulate a hypertensive state) would result in different (higher) peak wall stress [59]. However, we specifically want to draw attention to cases where clinically-determined geometric parameters, most notably the maximal AAA diameter, are beneath established thresholds for intervention but wall stress is elevated beyond a critical limit. We believe this analysis is more relevant under normotensive conditions where the impetus for surgical intervention is nominally lower, motivating our selection of the applied pressure boundary condition. Secondly, our simulations were carried out in the framework of classical continuum solid mechanics, and therefore ignore the potential for flow-induced shear stress on the inner vessel surface to impact AAA rupture risk, as pursued in previous studies [20,22,23,38,43,60]. Thirdly, the ultimate strength of vascular tissue is patient-specific and would vary with age and disease states, wherein our analyses assumed a single, representative ultimate strength of 1MPa based on the range of values found in the literature [36]. Finally, the ranges of geometries and material properties explored in our study are based on idealized scenarios rather than patient-specific characteristics, which limits the translational significance of our findings. However, rather than evaluating actual clinical scenarios, the aim of this study is to identify general (idealized) determinants of PWS that can be introduced into risk evaluation irrespective of patient-to-patient differences.

## Conclusion

The predicted interactive effects of sac diameter with thickness, axial extent, and material properties accentuate the need for integration of additional risk factors into clinical decision making in the context of AAA. Even with the few potential interactions considered in this study, it is evident that PWS values above the ultimate tissue strength are readily attainable in the sub-critical diameter range (< 5.0 cm). Taken together, our findings encourage an expansion of AAA parameters considered for clinical risk assessment and decision making, and demonstrate the potential for computational modeling to further elucidate key factors governing AAA mechanics.

## References

[1] Timaran CH, Veith FJ, Rosero EB, Modrall JG, Arko FR, Clagett GP, et al Endovascular aortic aneurysm repair in patients with the highest risk and in-hospital mortality in the United States. Arch Surg. United States; 2007;142: 520–525. doi: 10.1001/archsurg.142.6.520 17576887 10.1001/archsurg.142.6.520

[2] Ruddy JM, Jones JA, Ikonomidis JS. Pathophysiology of thoracic aortic aneurysm (TAA): is it not one uniform aorta? Role of embryologic origin. Prog Cardiovasc Dis. United States; 2013;56: 68–73. doi: 10.1016/j.pcad.2013.04.002 23993239 10.1016/j.pcad.2013.04.002 PMC3759819

[3] Nordon IM, Hinchliffe RJ, Loftus IM, Thompson MM. Pathophysiology and epidemiology of abdominal aortic aneurysms. Nat Rev Cardiol. England; 2011;8: 92–102. doi: 10.1038/nrcardio.2010.180 21079638 10.1038/nrcardio.2010.180

[4] Meijer CA, Stijnen T, Wasser MNJM, Hamming JF, van Bockel JH, Lindeman JHN. Doxycycline for stabilization of abdominal aortic aneurysms: a randomized trial. Ann Intern Med. United States; 2013;159: 815–823. doi: 10.7326/0003-4819-159-12-201312170-00007 24490266 10.7326/0003-4819-159-12-201312170-00007

[5] Baxter BT, Terrin MC, Dalman RL. Medical management of small abdominal aortic aneurysms. Circulation. United States; 2008;117: 1883–1889. doi: 10.1161/CIRCULATIONAHA.107.735274 18391122 10.1161/CIRCULATIONAHA.107.735274 PMC4148043

[6] Kent KC, Zwolak RM, Jaff MR, Hollenbeck ST, Thompson RW, Schermerhorn ML, et al Screening for abdominal aortic aneurysm: a consensus statement. J Vasc Surg. United States; 2004;39: 267–269. doi: 10.1016/j.jvs.2003.08.019 14718853 10.1016/j.jvs.2003.08.019

[7] Stroupe KT, Lederle FA, Matsumura JS, Kyriakides TC, Jonk YC, Ge L, et al Cost-effectiveness of open versus endovascular repair of abdominal aortic aneurysm in the OVER trial. J Vasc Surg. United States; 2012;56: 901–9.e2. doi: 10.1016/j.jvs.2012.01.086 22640466 10.1016/j.jvs.2012.01.086

[8] Danyi P, Elefteriades JA, Jovin IS. Medical therapy of thoracic aortic aneurysms. Trends Cardiovasc Med. United States; 2012;22: 180–184. doi: 10.1016/j.tcm.2012.07.017 22906366 10.1016/j.tcm.2012.07.017

[9] Sharma AK, Lu G, Jester A, Johnston WF, Zhao Y, Hajzus VA, et al Experimental abdominal aortic aneurysm formation is mediated by IL-17 and attenuated by mesenchymal stem cell treatment. Circulation. United States; 2012;126: S38–45. doi: 10.1161/CIRCULATIONAHA.111.083451 22965992 10.1161/CIRCULATIONAHA.111.083451 PMC3448933

[10] Vandy F, Upchurch GRJ. Endovascular aneurysm repair: current status. Circ Cardiovasc Interv. United States; 2012;5: 871–882. doi: 10.1161/CIRCINTERVENTIONS.111.966184 23250972 10.1161/CIRCINTERVENTIONS.111.966184

[11] Uchida N. Open stent grafting for complex diseases of the thoracic aorta: Clinical utility. Gen Thorac Cardiovasc Surg. 2013;61: 118–126. doi: 10.1007/s11748-012-0151-y 23054614 10.1007/s11748-012-0151-y PMC3589658

[12] Hinterseher I, Kuffner H, Berth H, Gabel G, Botticher G, Saeger HD, et al Long-term quality of life of abdominal aortic aneurysm patients under surveillance or after operative treatment. Ann Vasc Surg. Netherlands; 2013;27: 553–561. doi: 10.1016/j.avsg.2012.05.028 23540664 10.1016/j.avsg.2012.05.028

[13] Aljabri B, Al Wahaibi K, Abner D, Mackenzie KS, Corriveau M-M, Obrand DI, et al Patient-reported quality of life after abdominal aortic aneurysm surgery: a prospective comparison of endovascular and open repair. J Vasc Surg. United States; 2006;44: 1182–1187. doi: 10.1016/j.jvs.2006.08.015 17145419 10.1016/j.jvs.2006.08.015

[14] United Kingdom Small Aneurysm Trial Participants. Long-Term Outcomes of Immediate Repair Compared with Surveillance of Small Abdominal Aortic Aneurysms. N Engl J Med. 2002;346: 1445–1452. doi: 10.1056/NEJMoa013527 12000814 10.1056/NEJMoa013527

[15] Koskas F, Kieffer E. Long-term survival after elective repair of infrarenal abdominal aortic aneurysm: results of a prospective multicentric study. Association for Academic Research in Vascular Surgery (AURC). Ann Vasc Surg. Netherlands; 1997;11: 473–481. 9302059 10.1007/s100169900078

[16] Lund GB, Trerotola SO, Scheel PJJ. Percutaneous translumbar inferior vena cava cannulation for hemodialysis. Am J Kidney Dis. United States; 1995;25: 732–737. 7747727 10.1016/0272-6386(95)90549-9

[17] Weiford BC. Braunwald’s Heart Disease. The Journal of the American Medical Association. 2005.

[18] Dobrin PB. Pathophysiology and pathogenesis of aortic aneurysms. Current concepts. Surg Clin North Am. United States; 1989;69: 687–703. 2665139 10.1016/s0039-6109(16)44876-0

[19] Humphrey JD. Cardiovascular Solid Mechanics: Cells, Tissues, and Organs [Internet]. Springer New York; 2013 Available: https://books.google.com/books?id=gwbSBwAAQBAJ

[20] Georgakarakos E, Ioannou C V, Kamarianakis Y, Papaharilaou Y, Kostas T, Manousaki E, et al The Role of Geometric Parameters in the Prediction of Abdominal Aortic Aneurysm Wall Stress. Eur J Vasc Endovasc Surg. Elsevier Ltd; 2010;39: 42–48. doi: 10.1016/j.ejvs.2009.09.026 19906549 10.1016/j.ejvs.2009.09.026

[21] Giannoglou G, Giannakoulas G, Soulis J, Chatzizisis Y, Perdikides T, Melas N, et al Predicting the Risk of Rupture of Abdominal Aortic Aneurysms by Utilizing Various Geometrical Parameters: Revisiting the Diameter Criterion. Angiology. 2006;57: 487–494. doi: 10.1177/0003319706290741 17022385 10.1177/0003319706290741

[22] Chauhan SS, Gutierrez CA, Thirugnanasambandam M, De Oliveira V, Muluk SC, Eskandari MK, et al The Association Between Geometry and Wall Stress in Emergently Repaired Abdominal Aortic Aneurysms. Ann Biomed Eng. 2017;45: 1908–1916. doi: 10.1007/s10439-017-1837-1 28444478 10.1007/s10439-017-1837-1 PMC5529246

[23] Raghavan ML, Kratzberg J, Castro de Tolosa EM, Hanaoka MM, Walker P, da Silva ES. Regional distribution of wall thickness and failure properties of human abdominal aortic aneurysm. J Biomech. 2006;39: 3010–6. doi: 10.1016/j.jbiomech.2005.10.021 16337949 10.1016/j.jbiomech.2005.10.021

[24] Gasser TC, Auer M, Labruto F, Swedenborg J, Roy J. Biomechanical rupture risk assessment of abdominal aortic aneurysms: Model complexity versus predictability of finite element simulations. Eur J Vasc Endovasc Surg. Elsevier Ltd; 2010;40: 176–185. doi: 10.1016/j.ejvs.2010.04.003 20447844 10.1016/j.ejvs.2010.04.003

[25] Raghavan ML, Vorp DA, Federle MP, Makaroun MS, Webster MW. Wall stress distribution on three-dimensionally reconstructed models of human abdominal aortic aneurysm. J Vasc Surg. 2000;31: 760–9. doi: 10.1067/mva.2000.103971 10753284 10.1067/mva.2000.103971

[26] Vorp DA, Vande Geest JP. Biomechanical determinants of abdominal aortic aneurysm rupture. Arterioscler Thromb Vasc Biol. 2005;25: 1558–1566. doi: 10.1161/01.ATV.0000174129.77391.55 16055757 10.1161/01.ATV.0000174129.77391.55

[27] Fujikura K, Luo J, Gamarnik V, Pernot M, Fukumoto R, Tilson MD 3rd, et al A novel noninvasive technique for pulse-wave imaging and characterization of clinically-significant vascular mechanical properties in vivo. Ultrason Imaging. England; 2007;29: 137–154. doi: 10.1177/016173460702900301 18092671 10.1177/016173460702900301

[28] Nanayakkara BG, Gunarathne CK, Sanjeewa A, Gajaweera KAR. Geometric anatomy of the aortic- common iliac bifurcation. Gall Med J. 2007;12: 8–12.

[29] Holzapfel GA, Gasser TC, Ogden RW. A new constitutive framework for arterial wall mechanics and a comparative study of material models. J Elast. 2000;61: 1–48. doi: 10.1023/A:1010835316564

[30] Gasser TC, Ogden RW, Holzapfel G a. Hyperelastic modelling of arterial layers with distributed collagen fibre orientations. J R Soc Interface. 2006;3: 15–35. doi: 10.1098/rsif.2005.0073 16849214 10.1098/rsif.2005.0073 PMC1618483

[31] Weisbecker H, Pierce DM, Regitnig P, Holzapfel GA. Layer-specific damage experiments and modeling of human thoracic and abdominal aortas with non-atherosclerotic intimal thickening. J Mech Behav Biomed Mater. Elsevier Ltd; 2012;12: 93–106. doi: 10.1016/j.jmbbm.2012.03.012 22659370 10.1016/j.jmbbm.2012.03.012

[32] Pierce DM, Maier F, Weisbecker H, Viertler C, Verbrugghe P, Famaey N, et al Human thoracic and abdominal aortic aneurysmal tissues: Damage experiments, statistical analysis and constitutive modeling. J Mech Behav Biomed Mater. Elsevier; 2015;41: 92–107. doi: 10.1016/j.jmbbm.2014.10.003 25460406 10.1016/j.jmbbm.2014.10.003

[33] Bottasso CL, Detomi D. A procedure for tetrahedral boundary layer mesh generation. Eng Comput. 2002;18: 66–79. doi: 10.1007/s003660200006

[34] Tran AP, Fang Q. Fast and high-quality tetrahedral mesh generation from neuroanatomical scans. 2000; 1–20.

[35] Maas SA, Ellis BJ, Ateshian GA, Weiss JA. FEBio: Finite Elements for Biomechanics. J Biomech Eng. 2012;134: 11005 doi: 10.1115/1.4005694 22482660 10.1115/1.4005694 PMC3705975

[36] Raghavan ML, Webster MW, Vorp DA. Ex vivo biomechanical behavior of abdominal aortic aneurysm: Assessment using a new mathematical model. Ann Biomed Eng. 1996;24: 573–582. doi: 10.1007/BF02684226 8886238 10.1007/BF02684226

[37] Venkatasubramaniam AK, Fagan MJ, Mehta T, Mylankal KJ, Ray B, Kuhan G, et al A comparative study of aortic wall stress using finite element analysis for ruptured and non-ruptured abdominal aortic aneurysms. Eur J Vasc Endovasc Surg. 2004;28: 168–176. doi: 10.1016/j.ejvs.2004.03.029 15234698 10.1016/j.ejvs.2004.03.029

[38] Roy D, Holzapfel GA, Kauffmann C, Soulez G. Finite element analysis of abdominal aortic aneurysms: Geometrical and structural reconstruction with application of an anisotropic material model. IMA J Appl Math (Institute Math Its Appl. 2014;79: 1011–1026. doi: 10.1093/imamat/hxu037

[39] Fillinger MF, Raghavan ML, Marra SP, Cronenwett JL, Kennedy FE. In vivo analysis of mechanical wall stress and abdominal aortic aneurysm rupture risk. J Vasc Surg. 2002;36: 589–597. doi: 10.1067/mva.2002.125478 12218986 10.1067/mva.2002.125478

[40] Fillinger M. Who Should We Operate On and How Do We Decide: Predicting Rupture and Survival in Patients with Aortic Aneurysm. Semin Vasc Surg. 2007;20: 121–127. doi: 10.1053/j.semvascsurg.2007.04.001 17580250 10.1053/j.semvascsurg.2007.04.001

[41] Vande Geest JP, Wang DHJ, Wisniewski SR, Makaroun MS, Vorp DA. Towards a noninvasive method for determination of patient-specific wall strength distribution in abdominal aortic aneurysms. Ann Biomed Eng. 2006;34: 1098–1106. doi: 10.1007/s10439-006-9132-6 16786395 10.1007/s10439-006-9132-6

[42] Di Martino ES, Bohra A, Vande Geest JP, Gupta N, Makaroun MS, Vorp DA. Biomechanical properties of ruptured versus electively repaired abdominal aortic aneurysm wall tissue. J Vasc Surg. 2006;43: 570–576. doi: 10.1016/j.jvs.2005.10.072 16520175 10.1016/j.jvs.2005.10.072

[43] Rodríguez JF, Ruiz C, Doblaré M, Holzapfel GA. Mechanical stresses in abdominal aortic aneurysms: influence of diameter, asymmetry, and material anisotropy. J Biomech Eng. 2008;130: 21023 doi: 10.1115/1.2898830 18412510 10.1115/1.2898830

[44] Raghavan ML, Vorp DA. Toward a biomechanical tool to evaluate rupture potential of abdominal aortic aneurysm: Identification of a finite strain constitutive model and evaluation of its applicability. J Biomech. 2000;33: 475–482. doi: 10.1016/S0021-9290(99)00201-8 10768396 10.1016/s0021-9290(99)00201-8

[45] Rissland P, Alemu Y, Einav S, Ricotta J, Bluestein D. Abdominal Aortic Aneurysm Risk of Rupture: Patient-Specific FSI Simulations Using Anisotropic Model. J Biomech Eng. 2009;131: 31001 doi: 10.1115/1.3005200 19154060 10.1115/1.3005200

[46] Dorfmann A, Wilson C, Edgar ES, Peattie RA. Evaluating patient-specific abdominal aortic aneurysm wall stress based on flow-induced loading. Biomech Model Mechanobiol. 2010;9: 127–139. doi: 10.1007/s10237-009-0163-4 19578914 10.1007/s10237-009-0163-4

[47] Zarins CK, Xu C, Glagov S. Atherosclerotic enlargement of the human abdominal aorta. Atherosclerosis. 2001;155: 157–164. doi: 10.1016/S0021-9150(00)00527-X 11223437 10.1016/s0021-9150(00)00527-x

[48] Vorp DA. Biomechanics of abdominal aortic aneurysm. J Biomech. 2007;40: 1887–1902. doi: 10.1016/j.jbiomech.2006.09.003 17254589 10.1016/j.jbiomech.2006.09.003 PMC2692528

[49] Celi S., Berti S., Mariani M., Di Puccio F., Forte P. Investigation on the effect of the wall thickness in rupture risk estimation of aaa by a probabilistic finite element approach. Eur Heart J. 2010;31: 284.

[50] Vorp DA, Raghavan ML, Webster MW. Mechanical wall stress in abdominal aortic aneurysm: Influence of diameter and asymmetry. J Vasc Surg. 1998;27: 632–639. doi: 10.1016/S0741-5214(98)70227-7 9576075 10.1016/s0741-5214(98)70227-7

[51] Doyle BJ, Callanan A, Burke PE, Grace PA, Walsh MT, Vorp DA, et al Vessel asymmetry as an additional diagnostic tool in the assessment of abdominal aortic aneurysms. J Vasc Surg. Elsevier Inc.; 2009;49: 443–454. doi: 10.1016/j.jvs.2008.08.064 19028061 10.1016/j.jvs.2008.08.064 PMC2666821

[52] Scotti CM, Shkolnik AD, Muluk SC, Finol E a. Fluid-structure interaction in abdominal aortic aneurysms: effects of asymmetry and wall thickness. Biomed Eng Online. 2005;4: 64 doi: 10.1186/1475-925X-4-64 16271141 10.1186/1475-925X-4-64 PMC1298313

[53] Stevens RRF, Grytsan A, Biasetti J, Roy J, Liljeqvist ML, Christian Gasser T. Biomechanical changes during abdominal aortic aneurysm growth. PLoS One. 2017;12 doi: 10.1371/journal.pone.0187421 29112945 10.1371/journal.pone.0187421 PMC5675455

[54] Del Corso L, Moruzzo D, Conte B, Agelli M, Romanelli a M, Pastine F, et al Tortuosity, kinking, and coiling of the carotid artery: expression of atherosclerosis or aging? Angiology. 1998;49: 361–371. doi: 10.1177/000331979804900505 9591528 10.1177/000331979804900505

[55] Sacks MS, Vorp DA, Raghavan ML, Federle MP, Webster MW. In vivo three-dimensional surface geometry of abdominal aortic aneurysms. Ann Biomed Eng. United States; 1999;27: 469–479. 10468231 10.1114/1.202

[56] Fillinger MF, Marra SP, Raghavan ML, Kennedy FE. Prediction of rupture risk in abdominal aortic aneurysm during observation: Wall stress versus diameter. J Vasc Surg. 2003;37: 724–732. doi: 10.1067/mva.2003.213 12663969 10.1067/mva.2003.213

[57] Papaharilaou Y, Ekaterinaris JA, Manousaki E, Katsamouris AN. A decoupled fluid structure approach for estimating wall stress in abdominal aortic aneurysms. J Biomech. 2007;40: 367–377. doi: 10.1016/j.jbiomech.2005.12.013 16500664 10.1016/j.jbiomech.2005.12.013

[58] Pappu S, Dardik A, Tagare H, Gusberg RJ. Beyond Fusiform and Saccular: A Novel Quantitative Tortuosity Index May Help Classify Aneurysm Shape and Predict Aneurysm Rupture Potential. Ann Vasc Surg. 2008;22: 88–97. doi: 10.1016/j.avsg.2007.09.004 18023556 10.1016/j.avsg.2007.09.004

[59] Giannakoulas G, Giannoglou G, Soulis J, Farmakis T, Papadopoulou S, Parcharidis G, et al A computational model to predict aortic wall stresses in patients with systolic arterial hypertension. Med Hypotheses. 2005;65: 1191–1195. doi: 10.1016/j.mehy.2005.06.017 16107302 10.1016/j.mehy.2005.06.017

[60] Vande Geest JP, Schmidt DE, Sacks MS, Vorp DA. The effects of anisotropy on the stress analyses of patient-specific abdominal aortic aneurysms. Ann Biomed Eng. 2008;36: 921–932. doi: 10.1007/s10439-008-9490-3 18398680 10.1007/s10439-008-9490-3 PMC2674610

